# Antioxidant flavonoid-loaded nano-bioactive glass bone paste: *in vitro* apatite formation and flow behavior

**DOI:** 10.1039/d3na00941f

**Published:** 2024-01-05

**Authors:** Mehri Sohrabi, Saeed Hesaraki, Mostafa Shahrezaee, Alireza Shams-Khorasani, Fahimeh Roshanfar, Brigit Glasmacher, Sascha Heinemann, Yi Xu, Pooyan Makvandi

**Affiliations:** a Nanotechnology and Advanced Materials Department, Materials and Energy Research Center Alborz Iran Mehr.sohrabi@yahoo.com; b Trauma Research Center Tehran Iran Moshahrezayee@yahoo.com; c Institute for Multiphase Processes (IMP), Leibniz University Hannover 30823 Garbsen Germany; d Lower Saxony Centre for Biomedical Engineering, Implant Research and Development (NIFE) 30625 Hannover Germany; e INNOTERE GmbH Meissner Str. 191 01445 Radebeul Germany; f Department of Science & Technology, Department of Urology, NanoBioMed Group, The Quzhou Affiliated Hospital of Wenzhou Medical University, Quzhou People's Hospital Quzhou China drfelixxu@gmail.com yixu@wmu.edu.cn; g The Quzhou Affiliated Hospital of Wenzhou Medical University, Quzhou People's Hospital 324000 Quzhou Zhejiang China; h Centre of Research Impact and Outcome, Chitkara University Institute of Engineering and Technology, Chitkara University Rajpura-140401 Punjab India; i Department of Biomaterials, Saveetha Dental College and Hospitals, SIMATS, Saveetha University Chennai 600077 India

## Abstract

Non-cement pastes in the form of injectable materials have gained considerable attention in non-invasive regenerative medicine. Different osteoconductive bioceramics have been used as the solid phase of these bone pastes. Mesoporous bioactive glass can be used as an alternative bioceramic for paste preparation because of its osteogenic qualities. Plant-derived osteogenic agents can also be used in paste formulation to improve osteogenesis; however, their side effects on physical and physicochemical properties should be investigated. In this study, nano-bioactive glass powder was synthesized by a sol–gel method, loaded with different amounts of quercetin (0, 100, 150, and 200 μM), an antioxidant flavonoid with osteogenesis capacity. The loaded powder was then homogenized with a mixture of hyaluronic acid and sodium alginate solution to form a paste. We subsequently evaluated the rheological behavior, injectability, washout resistance, and *in vitro* bioactivity of the quercetin-loaded pastes. The washout resistance was found to be more than 96% after 14 days of immersion in simulated body fluid (SBF) as well as tris-buffered and citric acid-buffered solutions at 25 °C and 37 °C. All pastes exhibited viscoelastic behavior, in which the elastic modulus exceeded the viscous modulus. The pastes displayed shear-thinning behavior, in which viscosity was more influenced by angular frequency when the quercetin content increased. Results indicated that injectability was much improved using quercetin and the injection force was in the range 20–150 N. Following 14 days of SBF soaking, the formation of a nano-structured apatite phase on the surfaces of quercetin-loaded pastes was confirmed through scanning electron microscopy, X-ray diffractometry, and Fourier-transform infrared spectroscopy. Overall, quercetin, an antioxidant flavonoid osteogenic agent, can be loaded onto the nano-bioactive glass/hyaluronic acid/sodium alginate paste system to enhance injectability, rheological properties, and bioactivity.

## Abstract 2

Non-cement pastes in the form of injectable materials have gained considerable attention in non-invasive regenerative medicine.

## Graphical abstract

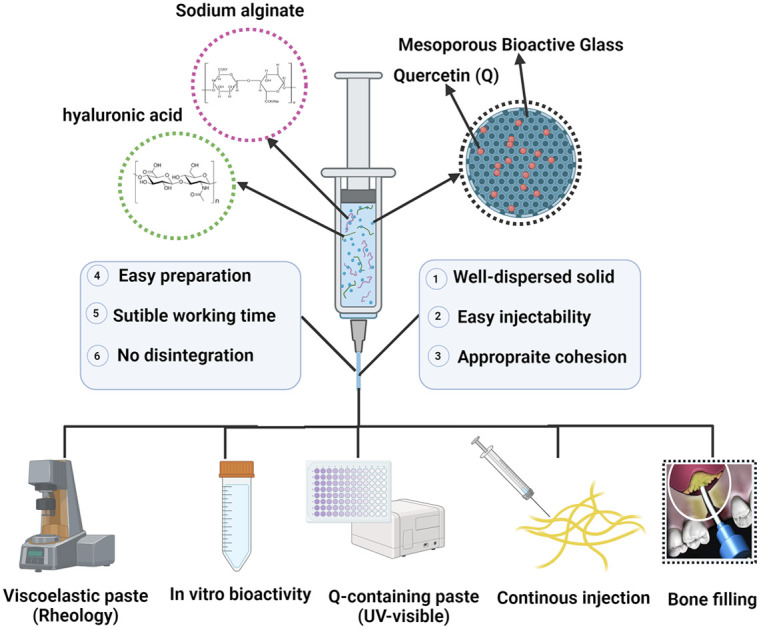

## 1. Introduction

Today, repairing bone defects caused by tumors, bone cysts, and fractures is still a challenge for surgeons;^1^ hence, much research is carried out in this field to regenerate bone defects and diseases using orthopedic biomaterials and implants.^2^ Among the types of materials used in this field, biomaterials such as hydroxyapatite, tricalcium phosphate, bioactive glass (BG), and calcium phosphate cement have attracted much attention owing to their biocompatibility and bioactivity.^3,4^ All these materials are available as powders, granules, and blocks; however, using them in the form of injectable pastes has many advantages in repairing bone defects using minimally invasive techniques.^5,6^

Although injectable cements have shown high potential for repairing bone defects, they possess limitations in clinical applications, including constraints related to injection time.^7^ According to previous studies, adding a polymeric phase also improved the injectability of cement.^8,9^ In another research study, glycerol was added to cement to enhance injectability; however, it extended the setting time.^10^ On the one hand, a long setting reaction is disadvantageous because an unset cement cannot withstand local stresses and consequently, can cause inflammatory response from disintegration.^11^ On the other hand, a fast setting reaction reduces the injectability of paste.^12^ Injectability is significant for minimally invasive surgery in filling bone defects and cracks through percutaneous vertebroplasty.^5,13,14^ Injectable bioactive materials are good candidates for filling bone defects and stabilizing osteoporotic bone.^14^ In addition, injectable bone filling materials are suitable options for treating dental root canal and other defects, which have small holes and restricted accessibility.^15^ Previously, bioactive pastes made of a bioactive solid phase and aqueous solutions of biocompatible natural polymers that do not undergo a cement setting reaction were developed.^16^ Composites based on an inorganic bioactive phase can react with body fluids and convert to hydroxyapatite, creating a strong bond with hard and soft tissues.^17,18^ The prospect of mixing polymer as a liquid phase with bioactive inorganic materials as a solid phase has sparked interest in tissue regeneration.^16,19,20^ A lot of attention has been directed toward employing these polymers in the development of injectable biomaterials due to the suitable interaction of bioactive inorganic particles and hyaluronate or sodium alginate (Alg) for the formation of injectable homogeneous compounds.^16,21^

The linear polysaccharide hyaluronate (Hac) is created by repeating units of glucuronic acid (GlcUA)-(1,3)-*N*-acetylglucosamine.^22^ As one of the main components of the extracellular matrix, it encourages cellular migration and proliferation.^23^ Hac is a water-soluble molecule that forms a gel to act as a lubricant. The adsorbing water makes it hygroscopic and homeostatic.^24^ This may be advantageous in some applications, such as orthopedic surgery, however, more structural stability and chemical stabilization are required for tissue engineering.^24,25^ The length of the chains (and the degree of entanglement), cross-linking, pH, concentration, and chemical modification are important factors that influence gel viscosity.^25^ Hac has consistently been regarded as a source of polymeric solutions because of its outstanding biocompatibility and viscoelastic properties.^25^ Moreover, it contributes to the development of the extracellular matrix by serving as an organizing core that joins intricate intracellular aggregates.^26^ In a previous study, Hac was used as a polymeric solution to create composite injectable pastes with the addition of BG.^26^ Due to their bioactivity and osteoconductivity, the inclusion of BG permitted the creation of direct chemical linkages to surrounding tissues. The varied morphology of the BG combined with Hac affected the rheological properties of the produced composite pastes.^27^ BG particles with large pore volumes and small particle sizes cause high paste viscosity,^16^ which can occur as a result of the liquid phase penetrating the pores, reducing the amount of liquid that is accessible between the particles.^16,27^

Alginates are made up of (1–4) connected (M units) -d-mannuronic acid and its C-5 epimer, -l-guluronic acid (G units).^28^ Alginate is a good biomaterial candidate for regenerative medicine because of its hydrophilicity, biocompatibility, and biodegradability, as well as its comparatively inexpensive cost.^28,29^ Despite all its positive features, alginate has poor mechanical strength, bioactivity, and osteoconductivity; hence, BG can be used in combination with alginate to improve biological performance in terms of reactivity.^29^ BG can influence the differentiation of osteoblasts by raising the levels of the markers ALP, osteocalcin, and osteopontin, which regulate genes involved in the cell cycle and progression towards mature osteoblasts.^29^ Through ionic interactions between the guluronic acid groups, sodium alginate exhibits solubility in aqueous solutions and forms stable gels at room temperature when exposed to non-cytotoxic concentrations of cations, such as Ba^2+^ and Ca^2+^.^29,30^ By crosslinking under non-cytotoxic conditions, 3D structures can be produced, typically with living cells encapsulated within the gel.^31^ The *in vivo* biodegradation of calcium-crosslinked sodium alginate has been reported in several animal models.^31^ In calcium phosphate, adding sodium alginate increases the cohesion properties of the paste.^32^ An injectable bioactive paste with developed biocompatibility and cell-proliferating properties was generated by combining BG with sodium alginate solution.^27^ In previous research, it was shown that the mineral carrying capacity, injectability, acellular bioactivity in SBF, biodegradation, proliferation, and differentiation of hMSCs were all optimal in the pastes consisting of BG nanoparticles and Alg.^33^ Several calcium phosphate formulations incorporating Alg and Hac have recently been developed.^34,35^ The cement paste cohesiveness and injectability were both improved by the addition of Alg and Hac.^34,35^

Due to outstanding biocompatibility, bioactivity, and osteoinductivity, BG is one of the most promising artificial bone-repairing materials.^36^ Ions released by BG, particularly soluble silicon and calcium ions, have been shown to stimulate osteoprogenitor cells at the genetic level, promoting bone regeneration.^17^ The previous studies confirmed that the biodegradation rate of BG is significantly lower than the calcium phosphates.^36,37^ BG particles are appropriate alternatives for incorporation into natural polymeric solutions to form an injectable paste.^27^ Although the paste made of BG particles is highly biocompatible and osteoconductive, some osteogenic agents/ions can be employed to make them osteoinductive.^37^ It has been reported that injectable Sr-containing bioactive glasses significantly increased the expression of osteogenic genes and type I collagen.^38^ Compared to the Sr-free BG/chitosan composite, using Sr-doped BG increased osteogenesis, induced bone growth, and demonstrated greater bone-implant contact. Moreover, unlike a chitosan-Mg-free BG composite, the chitosan-Mg-containing BG composite improved cellular survival and ALP activity.^38^ It is well known that the Mg ion not only acts as a co-factor for enzymes that stabilize the structure of RNA and DNA, but also affects the phenotype of osteogenic cells and accelerates bone remodeling.^38,39^

Based on the authors' knowledge, there are no studies about natural osteogenic molecule incorporation into BG pastes. In this study, different concentrations of quercetin were added to pastes made of bioactive glass, Hac, and Alg. The present paper focuses on the effects of added quercetin on the injectability, rheological behaviors, and apatite formation ability of the pastes. The rheological investigation can be utilized to examine the paste injectability as well as its behavior under applied load. Parameters such as viscosity, dynamic modulus, shear stress, and their relationships to time are some evaluated rheological factors that are crucial to understanding the flow behavior of the material.

## 2. Materials and methods

### 2.1. Preparation of starting materials and characterization

Sol–gel-derived nano-bioactive glass (NBG) was prepared based on 64SiO_2_·31CaO·5P_2_O_5_ mol% as described previously.^40^ The raw material was purchased from Merck (Wetzlar, Germany). Quercetin (Q) was purchased from Sigma-Aldrich (St. Louis, MO, USA).

After the synthesis of BG, the phase composition of BG powder was characterized by X-ray diffractometry (XRD), with Cu-Kα radiation and X-ray wavelength of 1.54050 Å (Dron 8, Bourevestink, Russia), operated at 40 kV and 40 mA at a step size of 0.04 and a count time of 2 s per step. The morphology of BG powder was characterized by electron microscopy (SEM, TEScan, VeGa II, Czech Republic).

### 2.2. Preparation of Q-loaded bioglass powder and determining the loading efficacy

Here, 100, 150, and 200 μM solutions of quercetin in ethanol were employed to load the drug onto the BG powder. The solution was placed in a shaker-incubator for 24 h and then, 2 g of the BG powder was soaked in 10 mL of the solution in a transparent, sealed vial. The mixture was dried at ambient temperature. The optical densities (OD) of the filtered BG-containing solution and reference quercetin solution at 370 nm (UV visible PG instrument T 80+, UK) were determined. A calibration line was obtained based on the OD values of known quercetin concentrations. Then, the proportion of the drug loaded on the BG was indicated by determining the difference in concentrations of Q before and after exposure to the BG.

### 2.3. Preparation of pastes

**Fig. 1:**
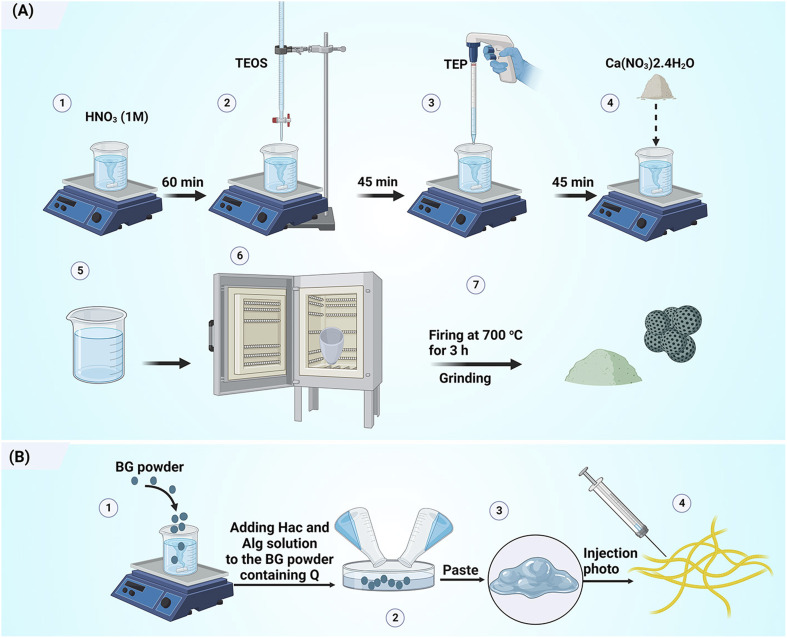
Schematic diagram illustrating the preparation of (A) quercetin-loaded mesoporous bioactive glass particles and (B) injectable bone paste.

A 3% solution of Hac and a 10% solution of Alg were prepared by dissolving the reagents in distilled water. The composites (suspensions of BG and the polymer solutions) were prepared by mixing quercetin-loaded NBG powder and the polymer solution at a powder-to-liquid ratio of 0.33 g mL^−1^ as shown in Table 1. The schematic diagram of the paste preparation is shown in Fig. 1.

**Table 1:** The paste formulations

Sample code	Solid phase	Liquid phase
PQ0	BG	3% solution of Hac and 10% solution of Alg
PQ100	100 μM Q-loaded BG	
PQ150	150 μM Q-loaded BG	
PQ200	200 μM Q-loaded BG	

### 2.4. *In vitro* bioactivity

The bioactivity of the pastes was assessed by immersion in Kokubo's simulated body fluid (SBF)^41^ for different intervals (1, 3, and 14 days). For this purpose, 1 g of each paste group was immersed in 30 ml SBF solution and stored at 37 °C. The SBF solution was renewed every 48 hours; at the end of each period, the paste was removed from the SBF solution, washed, and dried at room temperature. After that, the apatite formation ability was characterized by different methods.

The phase composition was determined by X-ray diffractometry (XRD) at the wavelength of 1.540 Å using a Philips PW3710 that operated at 20 kV and 10 mA. Data were collected at a step size of 0.05 and a count time of 2 s per step.

The change in morphology of the surfaces of the paste was visualised by scanning electron microscopy (SEM, Stereoscan S360, Cambridge) at an operating voltage of 20 kV and current intensity of 30 mA. The surfaces of the samples were gold-coated before the SEM observation.

Chemical characterization was carried out by Fourier Transform Infrared Spectroscopy (FTIR) in the wavelength range of 4000–400 cm^−1^, after sample preparation with KBr pellets.

### 2.5. Disintegration resistance

To determine the disintegration resistance (*D*_r_), the pastes were immersed in SBF solution for 1, 3, 7, and, 14 days. The *D*_r_ was calculated according to the following equation:^16^1
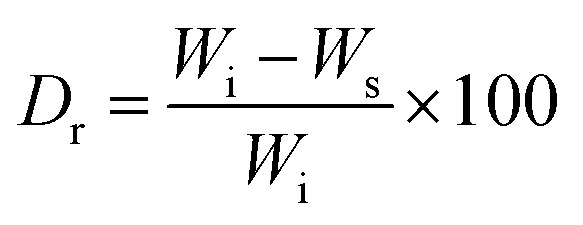
where *W*_i_ is the initial paste weight and *W*_s_ is the paste weight after the evaporation of the SBF solution.

The environmental sensitivity and stability of the pastes at different temperatures (*T* = 25 and 37 °C) and pH (5.4 and 7.4) conditions were evaluated by determining the *D*_r_ value and appearance of each paste. It should be noted that tris-buffered saline and citric acid-buffered solutions were selected for neutral and acidic environments, respectively.

### 2.6. Injectability

The injectability of the pastes was characterized based on 3 g of each paste extruded from a 3 ml syringe with tip outlets having diameters of 1.2 and 2.1 mm.

Extrusion was carried out using the SANTAM STM-20 tool at a feed rate of 30 mm min^−1^. Accordingly, the following eqn (2) was used for calculating the injectability (%).^8^2
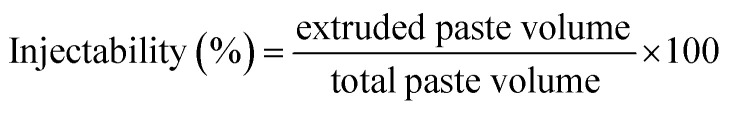


The injection curve was also determined as the injection force *vs.* the plunger displacement.

### 2.7. Rheological behaviors

The rheological properties in oscillatory mode were determined according to a previously described method.^16^ In brief, by using an Anton Paar MC-R300 rheometer with plate–plate geometry and a plate diameter of 25 mm, the rheology of pastes was measured in oscillation mode at room temperature. To determine the linear viscoelastic region, the loss modulus (*G*′′) and storage modulus (*G*′) were measured at various strains. The complex viscosity (η*) *versus* angular frequency (*ω*) at a constant strain (0.1%) was then measured. The time-sweep test was also determined to evaluate the change in viscosity of the pastes at a constant loading frequency.

## 3. Results and discussion

### 3.1. Structural characterization of NBG

**Fig. 2:**
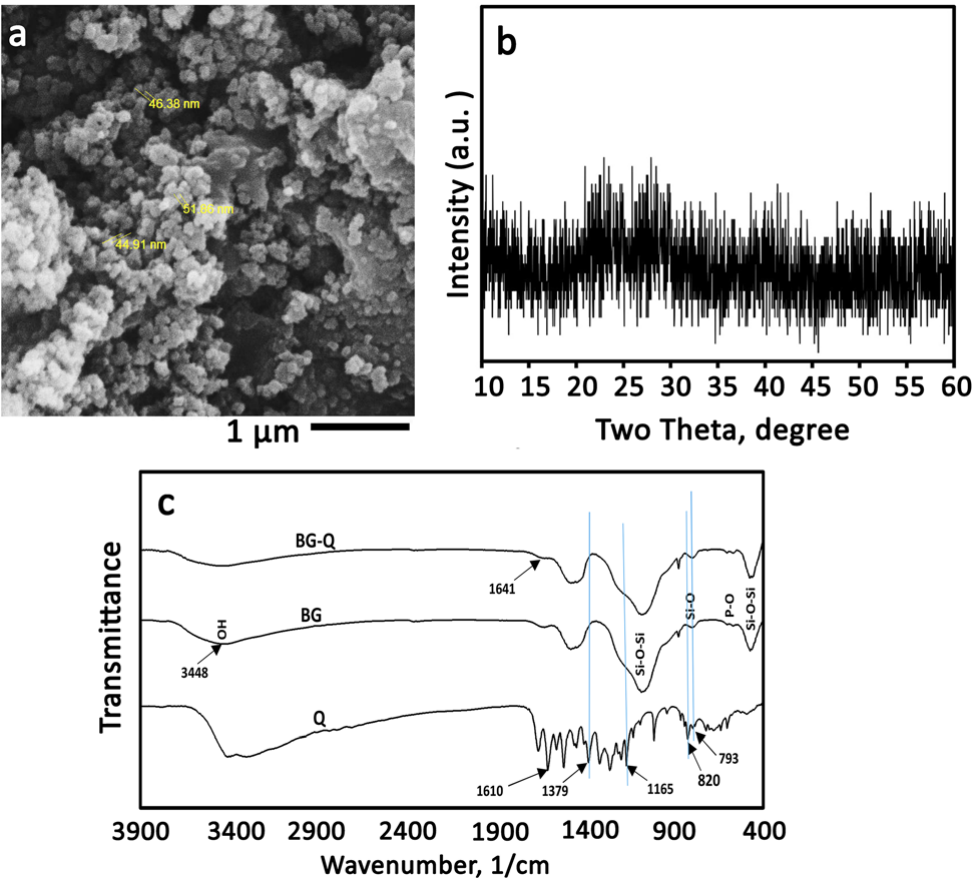
Structural characterization of the synthesized bioactive glass. (a) SEM image, (b) X-ray diffraction pattern, and (c) FTIR spectra of Q, BG, and Q-loaded BG.

NBG particles are attractive biomaterials for a variety of biomedical applications because of their too-small particle size, large specific surface area, and high surface-to-volume ratio.^42^ Additionally, because of its distinct morphology, it can be homogeneously combined with water-soluble polymers.^42^ In this study, the BG nanoparticles were synthesized according to our previous study.^27^ BG has a high specific surface area of 123 m^2^ g^−1^ and a total porosity of 0.60 cm^3^ g^−1^. Fig. 2a shows the SEM image of NBG powder. The morphology of the glass powder reveals porous agglomerated particles where the size of each particle is in the range of 40–100 nm. The synthesized NBG can be used as a drug and biomolecule carrier to regulate the release of these substances, due to their porosity and high specific surface area. Additionally, in the XRD pattern of the glass powder (Fig. 2b), no diffraction peak is observed, indicating its amorphous nature. The bioactivity and injection behavior can be strongly influenced by the BG particle size and porosity. Thus, they should be carefully controlled in biomedical applications. The rate of apatite precipitation in SBF solution is accelerated by the NBG particles. For applications like coating orthopedic implants, this influence is advantageous. Smaller BG particles also have a higher surface-to-volume ratio. BG is frequently used to repair and regenerate both hard and soft tissue due to its bone-bonding ability and osteogenesis properties. To increase the mechanical strength, stiffness, and dissolution rate, glass is often combined with other materials.^43^ Even though BG can intrinsically promote vascularization and bone regeneration, it is not always satisfactory, particularly for significant bone defects. For this reason, in this study, modification with quercetin was investigated as a further step to the addition of hyaluronic acid and sodium alginate polymers.

The chemical groups of quercetin, BG, and quercetin-NBG are depicted in Fig. 2c. All of the specific BG peaks are present in the spectra. Peaks at 1073, 879, and 475 cm^−1^ are due to with Si–O–Si bands. Peaks of the P–O band, which overlap with the Si–O–Si band at 1030 cm^−1^, were visible at 570 and 1030 cm^−1^. At 1470 and 3448 cm^−1^, OH chemical groups could also be seen.^16^ In the pure quercetin FTIR spectrum, OH group stretching was observed at 3406 and 3283 cm^−1^, and the OH bending group of the phenol was observed at 1379 cm^−1^.^16,44^ At 1666 cm^−1^, the C=O aryl ketonic stretching absorption was visible. Stretching bands for the C=C aromatic ring could be seen at 1610, 1560 cm^−1^, and 1510 cm^−1^. At 1317 cm^−1^, the C–H in-plane bending band of an aromatic hydrocarbon could be seen, and at 933, 820, 679, and 600 cm^−1^ there were out-of-plane bending bands. The stretching of the C–O atom in the aryl ether ring, the stretching of the C–O atom in phenol, and the stretching and bending of the C–CO–C atom in ketone, respectively, resulted in bands at 1263, 1200, and 1165 cm^−1^. However, the addition of quercetin to the NBG resulted in a slight reduction in the strength of these peaks and a downshift of the Si–OH bands, which were visible at 943 cm^−1^.^44^ These alterations may be related to the addition of quercetin to the inorganic matrix, which alters the length of the bonds in the SiO_2_ network and consequently, the bond strength. The formation of H-bonds involving the hydroxyl groups of the BG matrix was particularly suggested by the down-shifting of the Si–OH signal and the broadening of the OH band at 3448 cm^−1^. The same conclusion was also observed by Georgieva *et al.*^16,44^ On the other hand, the OH band associated with phenol widened and was intensified at 1379 cm^−1^. The stretching and bending of C–CO–C can be seen in the ketone band, as well as a slight broadening at 1165 cm^−1^. The low shift, broadening, and increase in the energy absorption of the OH bending band, as well as the slight broadening of the ketone bond, indicate that quercetin interacted with the OH groups of BG *via* a hydrogen bond that included the OH of phenol and C=O. The loss of continuity between aromatic rings resulted in an upshift of the aromatic ring C=C stretching band at 1610 cm^−1^ to a higher absorption value (1641 cm^−1^). The out-of-plane aromatic bending vibrations at 820 and 793 cm^−1^ changed, as did the vibrations at 825 and 797 cm^−1^.

It is suggested that the Si–O–Si band in the FTIR spectrum of the Q-loaded paste is broader than that of the Q-free paste. BG samples contain bands associated with flavonoids, and it is obvious that these bands are situated in the same locations as quercetin by enlarging the spectra in the range between 960 and 1730 cm^−1^ as displayed in Fig. 2c. The same was reported by Forte *et al.* who loaded quercetin on alendronate-functionalized hydroxyapatite.^45^

### 3.2. Quercetin loading

**Fig. 3:**
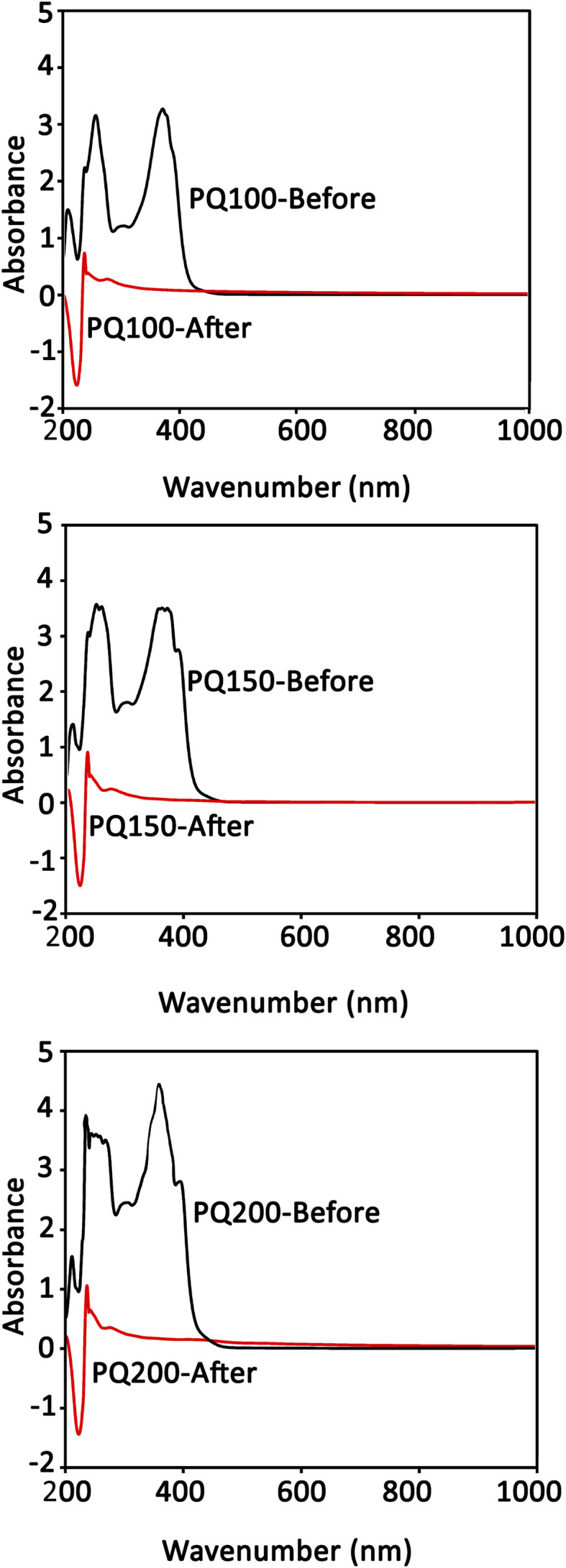
The quercetin UV absorption spectra of the loading media (ethanol) before and after the loading procedure.

The UV absorbance of the quercetin solution before and after loading on BG particles was determined. As shown by the UV curves in Fig. 3, the intensity of absorption peaks at 370 nm was reduced and nearly disappeared after loading. This means that the dissolved quercetin was adsorbed on the glass particles, and the loading process was successful. Note that the absorption intensity of the peak at 370 nm (associated with the quercetin) relates to the concentration of dissolved quercetin before loading. The results demonstrated a loading efficacy of >90% for all samples.

### 3.3. Injectability

**Fig. 4:**
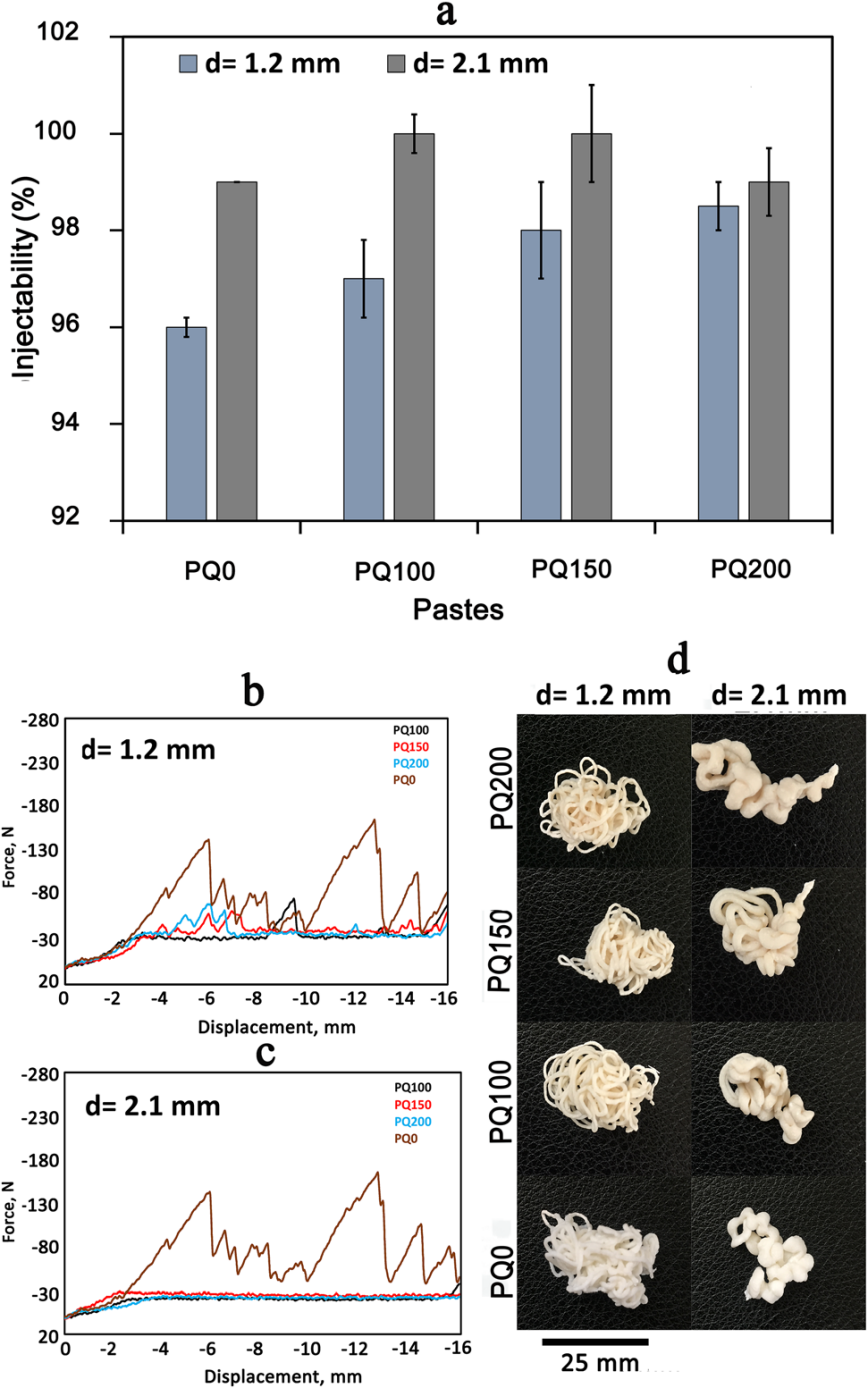
The injection test data: injectability percentage (a), injection curves (b and c), and the outward appearance of extruded pastes (d). Two different gauge diameters (1.2 mm and 2.1 mm) were employed for the injectability test.

Fig. 4(A) shows the injectability values of various pastes. Fig. 4(B) shows the syringe displacement, which is indicative of the current profile of the paste within the tube and cannula. The movement length was 16 mm and two different cannula diameters were employed as shown in Fig. 4(B-a) (syringe tip diameter of 1.2 mm) and Fig. 4(B-b) (syringe tip diameter of 2.1 mm). A complete paste injection was experienced using both cannula diameters, however different movement patterns were found. Unquestionably, greater injection force was needed as the diameter of the syringe outlet was reduced from 2.1 mm to 1.2 mm. Overall, the PQ0 showed teeth-like movement injection patterns from both tips, whereas the quercetin-containing paste revealed a nearly smooth pattern, indicating facile injection with a low extruding force. The authors suggest that the fluctuations in injection curves relate to the friction between the paste and the syringe wall, as well as the hydraulic pressure inside the syringe. This was also experienced in other studies.^46–48^ The high entanglement of glass particles (especially in PQ0) during the movement can be another reason. There is no quercetin in the PQ0, and the glass is mesoporous with high surface roughness. It seems that quercetin modifies the surface of the glass particles, causing less friction between the particles and improving flow during the injection. It was concluded that the drug molecules modify the glass surfaces, resulting in the easy movement of glass particles in the solution medium.

Fig. 4(C) shows the photographs of the paste injected from the syringes. All pastes were injected completely from both syringes with varying diameters. The shape of the pastes after injection is related to the syringe diameter, where the diameter of the extruded paste in the 2.1 mm syringe is wider than that from the 1.2 mm syringe. Although the paste was extruded with a higher force, no filter-pressing was observed.

### 3.4. Rheological behavior of the pastes in oscillatory mode

**Fig. 5:**
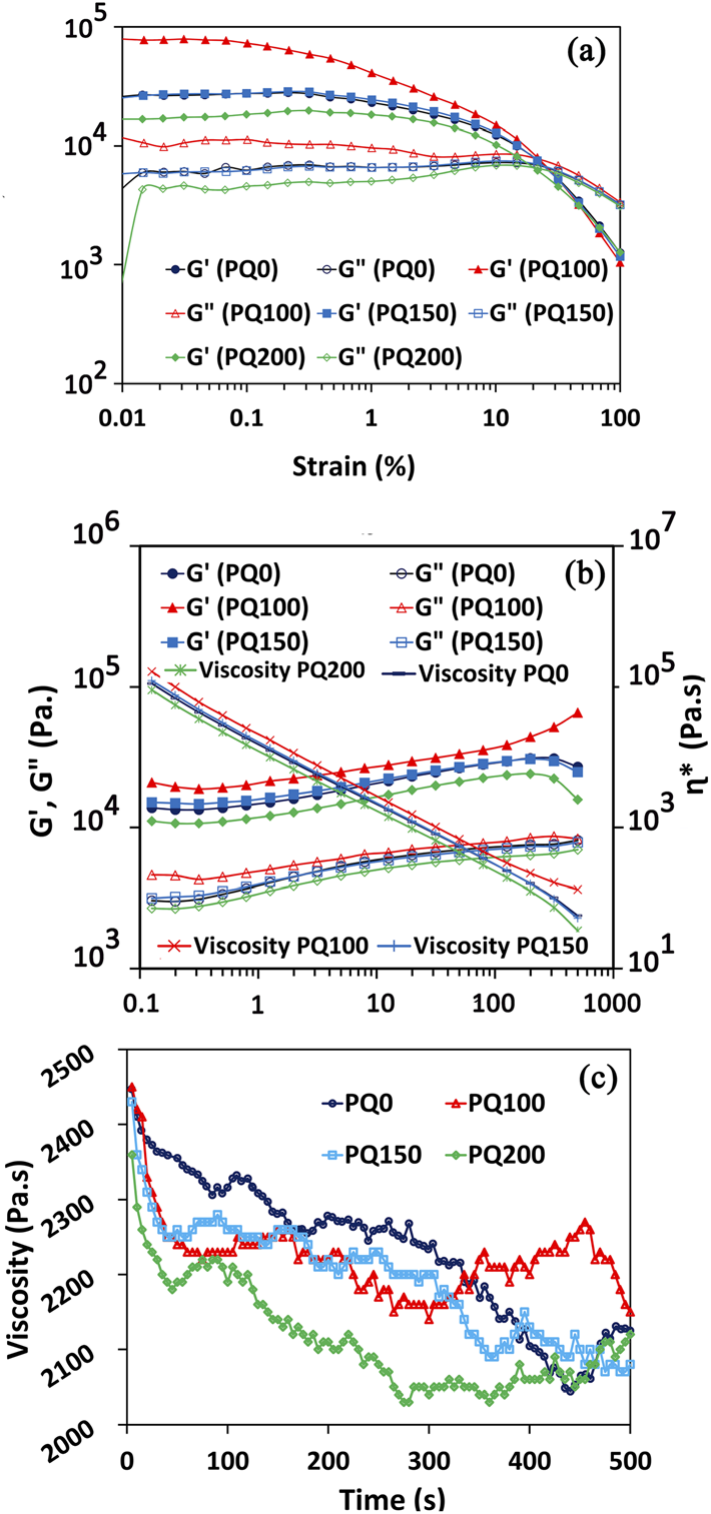
*G*′ and *G*′′ curves for all types of pastes in terms of strain (a), complex viscosity, *G*′, and *G*′′ in terms of angular frequency at constant strain (b), and viscosity as a function of time (c).

To determine the linear viscoelastic region of the paste, a strain sweep test was performed. Fig. 5a shows that as the strain percentage increases, the storage modulus (*G*′) is always greater than the loss modulus (*G*′′), and the distance between these two moduli is constant and in the form of a straight line. However, as the strain increased to a certain point, the distance between the two modules was gradually reduced and the positions of these two modules changed, the curves had a negative slope, and the loss modulus was greater than the storage modulus. The linear viscoelastic region extends from the point where these lines leave the flat state and begin to slope. The linear viscoelastic regions of various pastes are different. They occur at strains ≤1% for PQ200, ≤0.3% for PQ150, ≤0.1% for PQ100, and ≤0.3% for PQ0. Thus, the rheological measurements were performed at the constant strain of 0.1%, where all the pastes exhibited linear viscoelastic behavior.

Following the determination of the linear viscoelastic region, the complex viscosity, *G*′, and *G*′′ (in terms of angular frequency at constant strain), were analyzed (Fig. 5b). The viscosity decreased as the angular frequency increased, indicating the shear-thinning behavior of the pastes. All the frequencies, viscosities, loss moduli, and storage moduli of pastes PQ150 and PQ0 were nearly identical, while PQ200 had the lowest values. Remarkably, the elastic and loss modulus of the BG paste increased with the first addition of quercetin, *i.e.* 100 μM, while further addition decreased the modulus. Moreover, at all frequencies, the elastic moduli of all pastes were higher than the viscous moduli, indicating that the elastic nature overcomes the viscous nature. It is suggested that the surface modification of glass particles by the quercetin molecules through OH interactions of drug (Q) molecules with the Si–O– groups of the glass surface increased the paste modulus and viscosity. Another suggestion is the formation of chelates between the Ca^2+^ ions released from glass particles and the OH^−^ groups of partially delivered quercetin molecules.^45,49^ It is proposed that most Si–O– sites of the glass particles become involved through the OH groups of the quercetin drug, and the excess drug may be present as free non-interacting molecules. This would lead to a decrease in the modulus and viscosity by the easy slipping of free drug molecules. Hydrophobic drugs, such as quercetin, can also be included in BG by adjusting the drug–surface interactions. The availability of an appropriate silanol group on the surface of the BG particle, the –COOH or –OH group of the Q drug may be coupled easily *via* van der Waals forces or hydrogen bonding.^16,50^ Moreover, the hydroxyl groups in Q molecules have the potential for hydrogen bond formation along with the Si–OH and P–OH groups of BG.^16,45^

It is our opinion that in PQ100, the Q can be located in the glass particle pores. Hence, the high viscosity was only caused by the interaction of glass particles. However, by raising the Q concentration to 150 and 200 μM, the excess drug can cover the glass surface particles and facilitate movement when the frequency is applied. As a result, the viscosity is decreased gradually as the drug concentration increases.

Also, as shown in Fig. 5b, the elastic modulus is higher than the viscous modulus in all pastes and at all frequencies, meaning that the elastic nature of the pastes dominates the viscous nature. The PQ100 has the highest loss (viscous) modulus and storage (elastic) modulus, whereas the PQ200 has the lowest. Up to a frequency of 199, the elastic moduli (*G*′) of all pastes increased slowly (almost linearly), but beyond this frequency, except for PQ100, *G*′ exhibited a slight decrease. In the case of *G*′′, the same behavior was observed, *i.e.*, the viscous moduli of all pastes increased slowly (almost linearly) with frequency. Based on the complex viscosity curves, all pastes exhibited shear thinning behavior, which was indicated by a drop in viscosity as frequency increased. PQ100 has the highest viscosity and PQ200 has the lowest, while PQ100 and PQ150 are nearly equal in viscosity across all frequencies (their viscosity–frequency curves overlap).

Fig. 5c depicts the evolution of viscosity over time at a constant frequency. The viscosity of the pastes decreased over time, up to 500 seconds, indicating the ability of the pastes to flow. It was also demonstrated that PQ0 has the highest viscosity and PQ200 has the lowest viscosity.

### 3.5. *In vitro* disintegration and apatite formation ability

Table 2 lists the SBF disintegration resistance of the pastes at various immersion times. After 14 days of immersion, all samples retained their structures and no disintegration was observed. The paste disintegration would result in the movement of particles to the surrounding organs, causing tissue irritation or cytotoxic effects. In fact, by preserving the structure, an integrated ion-releasing matrix with stable surfaces for apatite precipitation is obtained.

**Table 2:** Paste disintegration resistance (%) at various immersion times in SBF

Time (day)	1	3	7	14
PQ0	99 ± 1	98 ± 2	97 ± 1	96 ± 2
PQ100	99 ± 1	99 ± 1	98 ± 1	96 ± 1
PQ150	99 ± 1	98 ± 2	97 ± 2	96 ± 2
PQ200	99 ± 1	99 ± 1	98 ± 1	97 ± 2

**Fig. 6:**
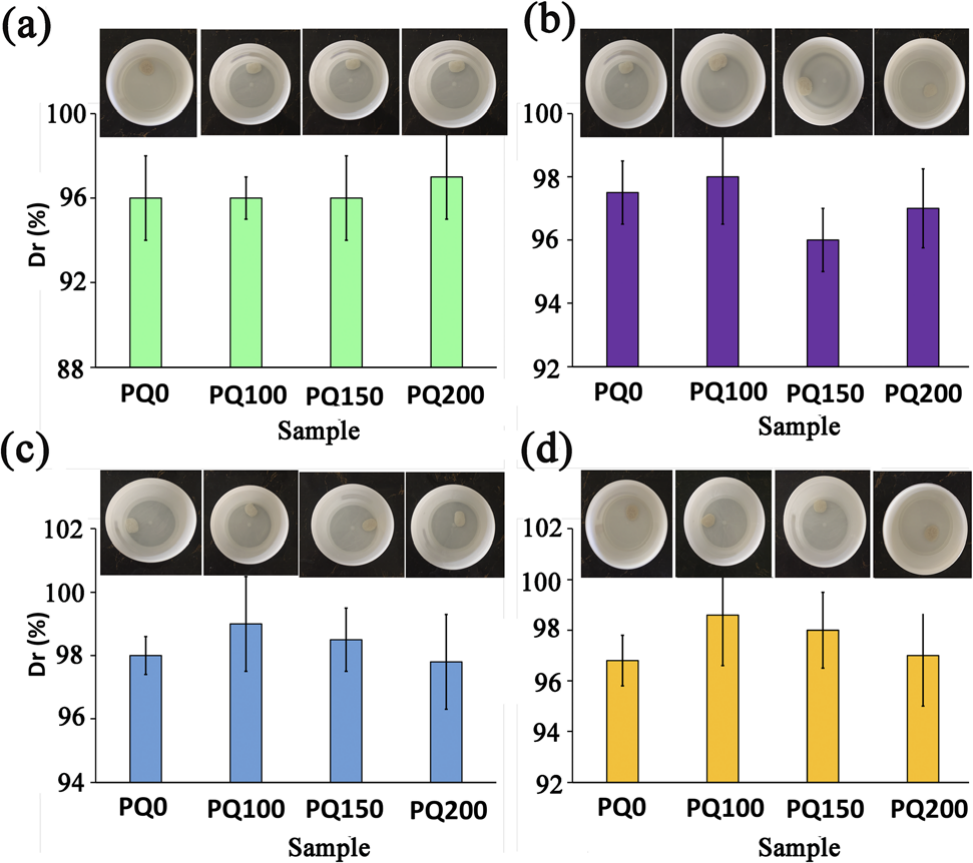
The disintegration resistance and outward appearance of the pastes at different pH and temperatures: (a): pH = 7.4, *T* = 25, (b): pH = 7.4, *T* = 37, (c): pH = 5.4, *T* = 25, and (d): pH = 5.4, *T* = 37.

The environmental sensitivity and stability of the pastes at different temperatures and pH conditions were also measured. Fig. 6 shows the disintegration resistance and outward appearance of the paste at different pH and temperatures (pH = 5.4 and *T* = 25, pH = 5.4 and *T* = 37, pH = 7.4 and *T* = 25, pH = 7.4 and *T* = 37) after immersion for 14 days in neutral and citric acid solutions. The results demonstrate that all pastes exhibited appropriate disintegration resistance (*D*_r_ > 96%) in different environmental situations and no washout phenomena were found. It suggests that the BG pastes have potential for bone repair in neutralized pH and acidic pH. A key hurdle during bone tissue regeneration is the excessive bacterial localization-induced acidic pH in the bone defect. The physicochemical properties of restorative/regenerative sealing materials are affected by the bacterial and inflammatory milieu with acidic pH in the bone defects. This justifies why evaluating the paste stability in acidic pH is essential. The washout resistance of the bioactive glass paste can be discussed by the formation of chelates between anionic polymers with a negative charge (alginate) and positive ions from the bioactive glass particles (Ca^2+^). In other words, the COO^−^ and OH^−^ functional groups of polymeric anions, hyaluronic acid and sodium alginate can interact with Ca^2+^ ions that exist on the surfaces of glass particles to form calcium hyaluronate and calcium alginate gels^51,52^ which are water-insoluble complexes and hinder the disintegration of BG paste in the physiological fluids. This mechanism occurs in both neutral and acidic pH and serves to maintain the paste's structural stability.

When the paste is soaked in SBF with neutralized pH, in addition to the above-mentioned mechanism, the disintegration resistance of the paste can also be attributed to the formation of an apatite layer on the paste surfaces. The mechanism of apatite formation on bioactive glass products has well been discussed in the literature.^20,27,40,47^ The capacity of bioactive glasses to release ions develops a silica-rich layer leading to a decrease in the pH. After that, Ca–P precipitation and further crystallization of the apatite phase occur, which raises the pH. The formation of an apatite phase on the surfaces of bioactive glass pastes can improve the paste's stability and disintegration resistance. In addition, because cell viability and cell differentiation can be damaged in acidic pH, the paste with the potential to neutralize acidic pH in the damaged bone tissue can guarantee cell-dependent processes.

**Fig. 7:**
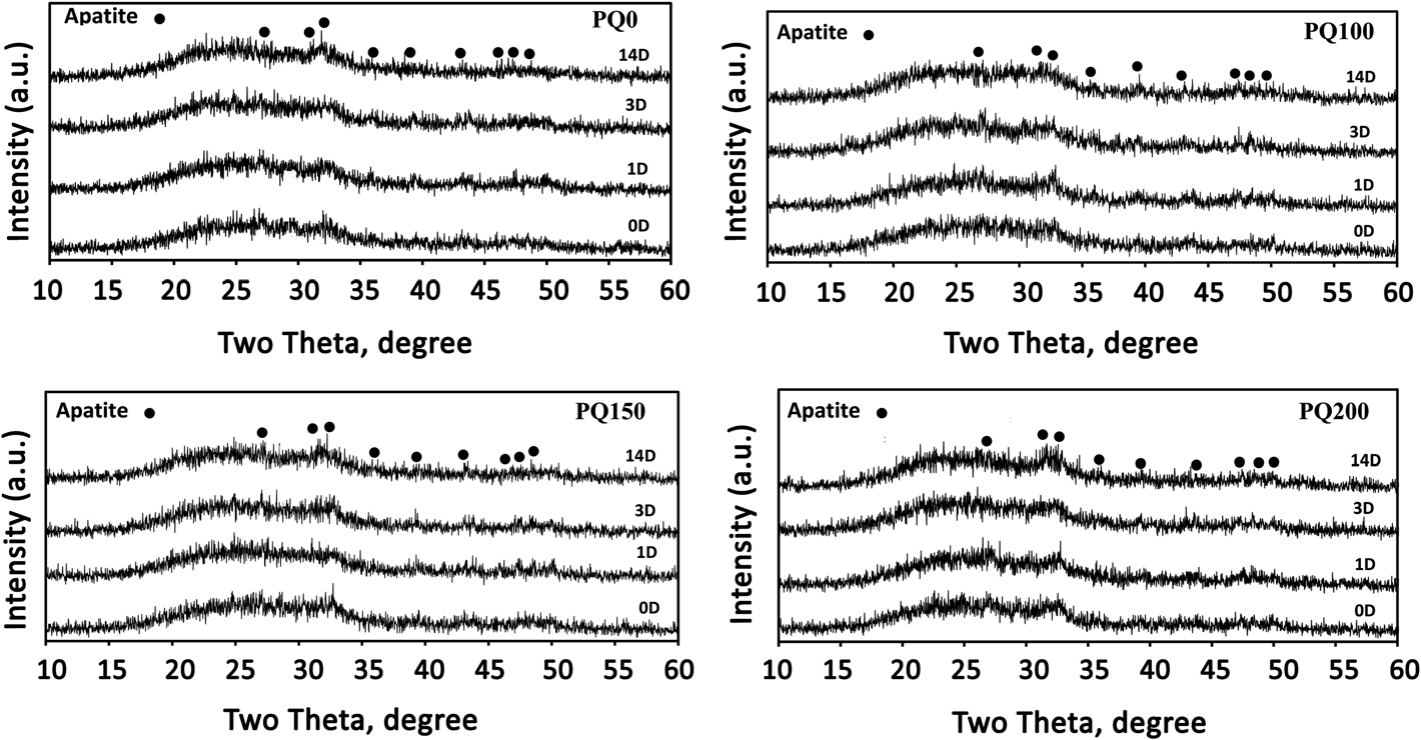
XRD patterns of the different pastes before and after immersion in SBF solution for 1, 3, and 14 days.

Fig. 7 shows the X-ray diffractometry patterns of the pastes before and after immersion in SBF solution. Because they can create a beneficial interface between the material surface and bone tissue, inorganic materials (bioactive ceramics) like hydroxyapatite or bioactive glasses (BG) have received a lot of interest.^53,54^ To generate a layer of hydroxycarbonate apatite (HCA), which is identical to the hydroxyapatite found naturally in bone, on the surface of these biomaterials, contact with body fluids is suggested for bone-bonding creation.^51–53,55^

Signs of a poorly crystalline apatite phase as a broad diffraction pattern are observed before the immersion of the pastes in SBF solution. The presence of an apatite phase before immersion in SBF can be due to the dissolution–precipitation phenomenon of the glass particles and apatite within the water phase of the paste. In other words, precipitation appears to be caused by a reaction between Ca^2+^ and PO_4_^3−^ ions released from the glass particles, followed by precipitation.^8,51^

It has been found that the intensity of apatite peaks increases by increasing the immersion time through the dissolution and release of calcium and phosphate ions from BG, as well as the precipitation and densification of the apatite phase. The XRD pattern (Fig. 7) shows the peaks at 28.89, 31.7, 32.86, 35.42, 39.7, 44.32, 46.66, 48.54, and 50.43, which are attributed to the crystalline apatite phase (210), (211), (300), (310), (130), (400), (222), (230) and (321) diffraction, respectively. Based on the XRD results, there is no considerable difference between the various samples, however, the PQ200 exhibits more intensive apatite peaks.

**Fig. 8:**
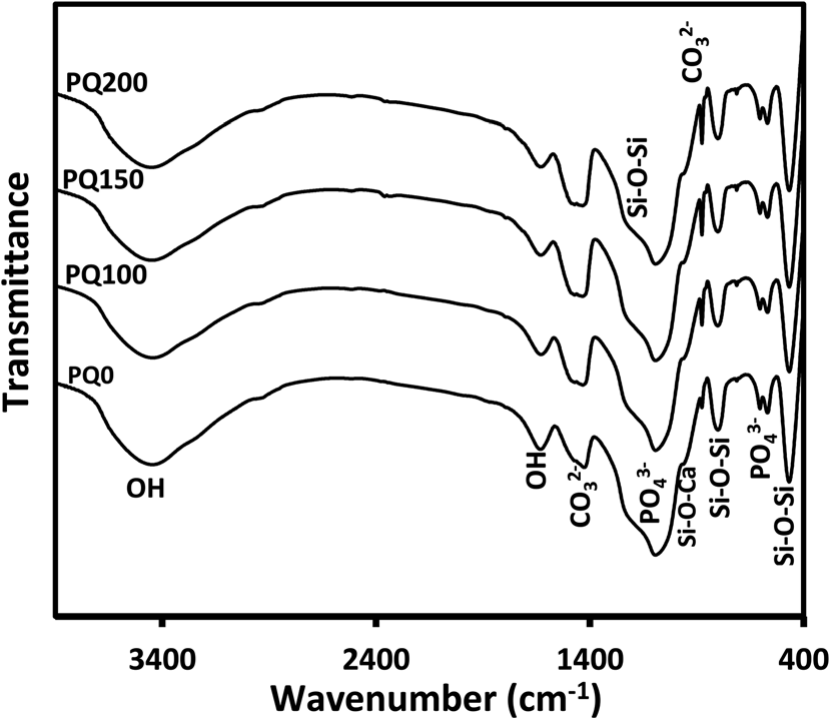
The Fourier transform infrared spectra of all paste types after 14 days of immersion in SBF solution.

After 14 days of immersion in SBF solution, Fig. 8 displays the FTIR results for all varieties of pastes, which support the XRD findings. The twin peaks observed at about 570 and 605 cm^−1^ were assigned to the P–O stretching of the apatite lattice.^26,39^ The broad Si–O–Si and P–O bands were observed at 1215 cm^−1^ and 1050 cm^−1^, respectively. The absorption bands at 1630 and 3000–3750 cm^−1^ were assigned to the OH group.^52,55,56^ The signals of the (CO_3_)^2−^ group substituted for the (PO_4_)^3−^ group in the apatite lattice were found at 870, 1420, and 1460 cm^−1^.^8,51,57^ When the Q content increased, the single C–O stretching band at 1420 cm^−1^ changed into a twin (at 1420 and 1460 cm^−1^), while the absorption intensity of the C–O band at 870 cm^−1^ increased.^45^

**Fig. 9:**
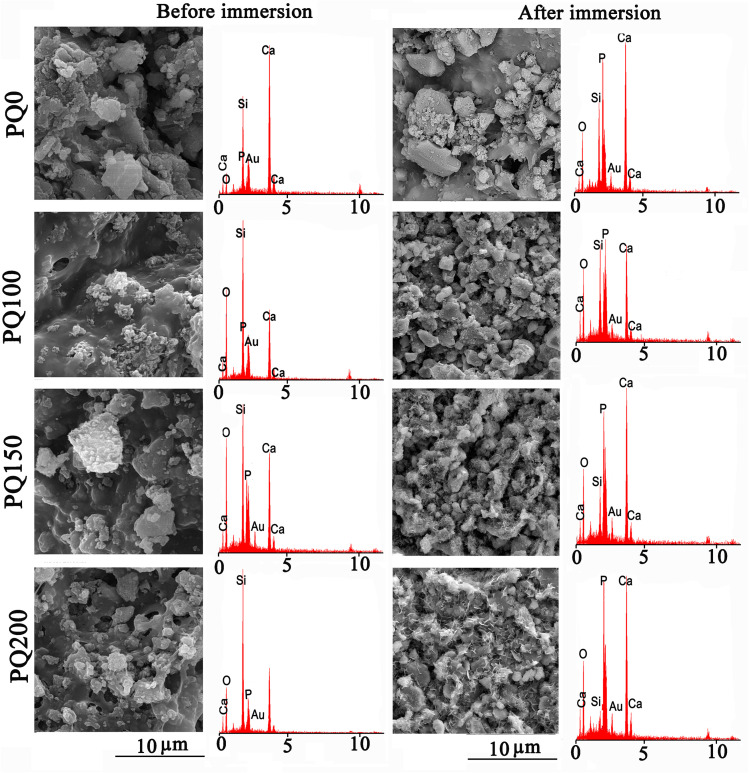
The scanning electron microscopy images and the related EDS patterns of various pastes before and after 14 days of soaking in SBF solution.

The SEM micrographs and their related EDS patterns of the pastes before and after 14 days of immersion in SBF solution are shown in Fig. 9. Before immersion, a monolithic phase, including fine aggregated particles was observed. Porosity was also observed due to the evaporation of the water phase. A higher content of pore volume was observed in the Q-free paste. After immersion, the leaching out of the monolithic phase occurred and disappeared, and was replaced by some micropores. In the quercetin-free sample, particles with rough surfaces were observed. In quercetin-loaded pastes, some precipitates having a flakelike network on the surfaces of the original glass particles were observed. It seems that the thickness and extent of the flakes increased in the microstructures of pastes with higher quercetin content.

According to the EDS analysis of the pastes, Si was the most abundant element on the surface before soaking. However, after soaking, the Si peak decreased and the Ca and P peak intensities improved because of precipitated calcium phosphate (poorly crystalline apatite) on the paste surface during the SBF immersion. The decrease in the intensity of the Si peak compared to Ca and P was more evident in samples containing quercetin. The EDS analysis confirmed that compared to PQ100, and PQ150, more calcium phosphate phase precipitated on the PQ200 surface. These observations are in line with the FTIR and XRD results.

A comparison of the XRD, FTIR, and SEM/EDS patterns before and after immersion in SBF revealed that the studied pastes can support apatite formation. This means that the nanoapatite phase precipitated on the surfaces of BG particles *via* the soaking procedure, and the extent of precipitation was influenced by the presence and content of quercetin.

The data strongly suggest that Q and BG may form a chemical bond within the particle framework. The interaction between OH groups of Q molecules and the Ca ions may be responsible for this interaction and phosphate adsorption resulting in calcium phosphate precipitation.^45,52,58^

## 4. Conclusion

The injectability, disintegration resistance, rheology, and *in vitro* behaviors of bioactive glass-hyaluronic acid-sodium alginate with various contents of loaded quercetin were investigated. The results demonstrated that quercetin affects apatite formation, injectability, and rheological properties. Moreover, quercetin improves the paste injectability by decreasing the viscosity. The produced pastes exhibit viscoelastic behavior in which the elastic nature overcomes the viscous one. Changes in viscosity, storage modulus, and loss modulus depend on the quercetin concentration. The composite bioactive glass-Hac-Alg paste exhibited apatite formation ability, and the extent of the precipitated apatite increased with the addition of quercetin. The study revealed that all pastes exhibited adequate disintegration resistance and anti-washout properties in different pH and temperature conditions. The present study focused on the injectability and bioactivity of designed quercetin-loaded bioactive glass-Hac-Alg paste, understanding that the osteogenic properties of the paste will help researchers estimate the efficacy in terms of functionality. Thus, the quercetin release behavior as well as the osteogenic gene expression of the loaded paste should be evaluated in further studies.

## Conflicts of interest

There are no conflicts to declare.

## Supplementary Material
